# Future Economics of Liver Transplantation: A 20-Year Cost Modeling Forecast and the Prospect of Bioengineering Autologous Liver Grafts

**DOI:** 10.1371/journal.pone.0131764

**Published:** 2015-07-15

**Authors:** Dany Habka, David Mann, Ronald Landes, Alejandro Soto-Gutierrez

**Affiliations:** 1 Health Systems Reform, Beirut, Lebanon; 2 Cellular Dynamics International, Madison, WI, United States of America; 3 Solving Organ Shortage, Austin, TX, United States of America; 4 Department of Pathology, University of Pittsburgh, Pittsburgh, PA, United States of America; 5 Thomas E. Starzl Transplantation Institute, University of Pittsburgh, Pittsburgh, PA, United States of America; 6 McGowan Institute for Regenerative Medicine, University of Pittsburgh, Pittsburgh, PA, United States of America; 7 SOS Whole Liver Research Community, Austin, TX, United States of America; ISMETT-UPMC Italy/ University of Catania, ITALY

## Abstract

During the past 20 years liver transplantation has become the definitive treatment for most severe types of liver failure and hepatocellular carcinoma, in both children and adults. In the U.S., roughly 16,000 individuals are on the liver transplant waiting list. Only 38% of them will receive a transplant due to the organ shortage. This paper explores another option: bioengineering an autologous liver graft. We developed a 20-year model projecting future demand for liver transplants, along with costs based on current technology. We compared these cost projections against projected costs to bioengineer autologous liver grafts. The model was divided into: 1) the epidemiology model forecasting the number of wait-listed patients, operated patients and postoperative patients; and 2) the treatment model forecasting costs (pre-transplant-related costs; transplant (admission)-related costs; and 10-year post-transplant-related costs) during the simulation period. The patient population was categorized using the Model for End-Stage Liver Disease score. The number of patients on the waiting list was projected to increase 23% over 20 years while the weighted average treatment costs in the pre-liver transplantation phase were forecast to increase 83% in Year 20. Projected demand for livers will increase 10% in 10 years and 23% in 20 years. Total costs of liver transplantation are forecast to increase 33% in 10 years and 81% in 20 years. By comparison, the projected cost to bioengineer autologous liver grafts is $9.7M based on current catalog prices for iPS-derived liver cells. The model projects a persistent increase in need and cost of donor livers over the next 20 years that’s constrained by a limited supply of donor livers. The number of patients who die while on the waiting list will reflect this ever-growing disparity. Currently, bioengineering autologous liver grafts is cost prohibitive. However, costs will decline rapidly with the introduction of new manufacturing strategies and economies of scale.

## Introduction

Approximately 30 million people in the U.S. have a liver disorder. About 40,000 of them will progress to end-stage liver disease, which is responsible for approximately 30,000 deaths annually in the U.S. [1, 2]. Medical therapy can extend life, but the only curative therapy for severe end-stage liver disease is allogeneic liver transplantation—either a partial liver from a living-related donor or a whole cadaveric liver. However, liver transplantation is severely limited by the supply of donors.

In many ways, liver transplantation has been a victim of its own success. As transplantation science evolved, indications for this therapeutic modality expanded to include many causes of acute and chronic liver failure, cirrhosis, inherited metabolic diseases and some cases of cancers [3]. Yet the pool of donor livers failed to keep pace with the growing demand; in some areas it is losing ground. In the U.S., the annual number of cadaveric donor livers decreased from 7,014 in 2006 to 5,798 in 2014, according to data collected by the Organ Procurement and Transplantation Network. Living donation numbers have also declined, falling from about 524 donors in 2001 to 230 in 2014 [4]. Of the 16,000 people on the liver waiting list in 2014, less than half of them (5,527) received a transplant [4]. To make matters worse, the donor pool is expected to shrink further due to the obesity epidemic. Liver steatosis is increasingly common in donors and is a significant risk factor in liver transplantation [5]. These data point to one fact: Organ availability is an absolute constraint on the number of liver transplants that can be performed.

Numerous unconventional strategies have been explored to increase the number of livers available for transplantation. These include: the use of marginal donors, an ill-defined group comprised of donors over the age of 60; donors with greater than 30% hypernatremia or macrosteatosis; donors with positive serologies for the hepatitis C or hepatitis B virus; donors with a cold ischemia time of greater than 12 hours; non-heart-beating donors; and grafts from split-livers or living-related donors [3, 6]. Resuscitation of marginal quality donor organs using machine perfusion [7] and the production of pigs with genetic manipulations [8] has also been explored. Unfortunately, these alternative approaches have presented a variety of practical and logistic difficulties.

Because the liver has the ability to regenerate completely after severe liver failure, it benefits from methods of temporary hepatic support and hepatocyte transplantation [9]. Auxiliary liver transplantation [10] has also developed as an effective therapeutic option for patients with acute liver failure and inborn errors of liver metabolism, though this approach has inherent limiting factors similar to orthotopic liver transplantation.

Creating a universally available liver graft from autologous tissue and cells would increase the number of organs available for transplantation and eliminate the need for a life-long regimen of immune suppression drugs and their complications. Clearly there is a compelling social need to perform liver transplantation more widely. Now that many of the scientific elements are falling into place, it will likely be possible to bioengineer a liver from autologous cells.

The transplant community faces major challenges treating end-stage liver disease—social, political and monetary. In this paper we attempt to define the size of the problem by creating an economic model for liver transplantation over the next 20 years. We projected the population in need plus the costs of treatment with conventional allogeneic liver transplantation. Then we looked at the prospective costs of transplanting livers bioengineered from autologous induced pluripotent stem cells (iPSCs).

## Methods

### Economic Model

Our model is divided into two parts: The Epidemiology Model and the Treatment Costs Model. The Epidemiology Model forecasts the population dynamics of liver transplantation: wait-listed patients; operated patients, and postoperative patients out to 10 years. A simulation period of 20 years is considered for the cohort of wait-listed patients. (Patients prior to 2014 or after 2033 are excluded).

**Fig 1:**
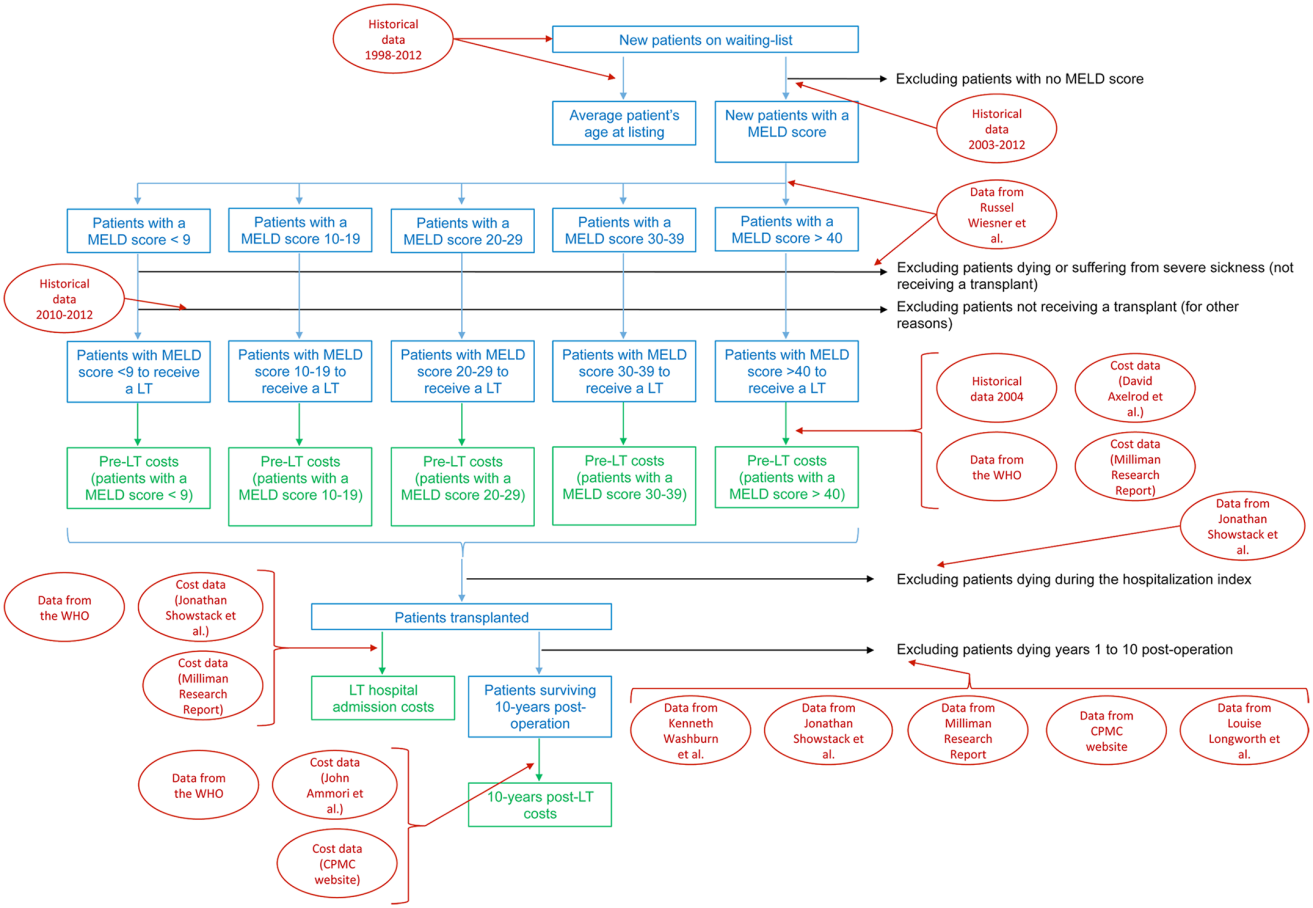
Model Scheme. Blue and green rectangles represent, respectively, the forecast outputs of the Epidemiology Model and the Treatment Costs Model. Data used to perform the forecasts are shown in red square (refer to Table 1 for further details on data source).

The Treatment Costs Model forecasts costs generated during the simulation period. Costs are divided into pre-transplant-related costs, transplant (admission)-related costs and 10-year post-transplant-related costs. The patient population has been categorized using the Model for End-Stage Liver Disease (MELD) score. The pre-transplant-related costs are allocated per MELD score (<9, 10–19, 20–29, 30–39, >40). They account for the costs generated one year prior to transplant (for severe illness) or while patients are on the waiting list (for moderate illness with waiting time exceeding one year). In this way we were able to capture the costs of treatment prior to transplantation for severely ill patients—who will be on the waiting list for days to a couple of months—compared to patients who are less ill and could be on the waiting list for up to five years. Due to the lack of published data, the admission-related costs and post-transplant-related costs are shown for all patients without regard to the MELD score. All costs are expressed in 2014 U.S. dollars (USD) and, where appropriate, were adjusted to 2014 USD using the Gross Domestic Product (GDP) deflator-based calculator [11]. The 20-years forecasted cost values were discounted to present value at a rate of 3% per year [12, 13]. Fig 1 shows the model scheme.

#### Forecast metrics

The outcomes of the Epidemiology Model are the number of new patients listed each year and their distributions based on MELD score and age at listing. Additionally, the following metrics are forecast: (Pre-liver transplantation) number of patients who will die or suffer severe illness on the waiting list; (Pre-liver transplantation) number of patients who will not receive a liver transplantation; (Pre-liver transplantation) number of patients who will receive a liver transplant; (Admission) number of patients surviving surgery; and (Post-liver transplantation) number of patients surviving Year 1 through Year 10 after liver transplantation.

Outcomes of the Treatment Cost Model are: 1) the discounted treatment costs during the pre-liver transplantation phase, per to-be transplanted patients and per MELD score; 2) the discounted total resources used from the day of transplantation to discharge, per transplant-operated patient; and 3) the discounted total costs of treatment 10 years post-liver transplantation per patients surviving for 10 years. All metrics are shown over the 20-year period of simulation and at Years 1, 10 and 20.

#### Uncertainty and sensitivity analyses

In numerical modeling, an uncertainty analysis employs different techniques for determining the reliability of model predictions and accounts for various sources of uncertainty in model input and design. In our model an uncertainty analysis is performed to generate plausible value ranges of the costs metrics at Year 20 (2033), and to assess the certainty at which the base case can be achieved. For this purpose, parameters are varied according to a realistic distribution and 30,000 Monte Carlo iterations are run using Oracle Crystal-Ball software. We then performed a sensitivity analysis, which determines how different values of an independent variable will impact the financial output metrics.

#### Data sources

The epidemiology and costs data are collected from various literature sources and are summarized in Table 1.

**Table 1:** Model inputs’ values and their associated distributions/ranges.

**Real patients’ cohort size and characteristics in year 2012**					
**Reference**	**Parameter**	**Distribution**	**Base**	**Low**	**High**
HHS-HRSA-SRTR [14]	New patients on waiting-list	Fixed	10,143	-	-
	Percent patients with not-defined MELD score	Fixed	0.094	-	-
	Percent patients with inactive status on waiting-list	Fixed	0.129	-	-
	Percent patients with MELD score 6–14	Fixed	0.432	-	-
	Percent patients with MELD score 15–34	Fixed	0.319	-	-
	Percent patients with MELD score > 35	Fixed	0.026	-	-
	Percent age group 18–34	Fixed	0.042	-	-
	Percent age group 35–49	Fixed	0.147	-	-
	Percent age group 50–64	Fixed	0.627	-	-
	Percent age group > 65	Fixed	0.184	-	-
**Theoretical patients’ cohort size and characteristics forecasts (General Epidemiology Model)**					
**Reference**	**Parameter**	**Distribution**	**Base**	**Low**	**High**
HHS-HRSA-SRTR [14] / Computed	Percent change new patients on waiting-list	Triangular	0.011	-0.014	0.079
	Percent change patients with not-defined MELD score	Triangular	0.076	0.049	0.094
	Percent change patients with inactive status on waiting list	Triangular	-0.005	-0.068	0.051
	Percent change patients with MELD score 6–14	Triangular	-0.027	-0.04	-0.006
	Percent change patients with MELD score 15–34	Triangular	0.022	0.002	0.046
	Percent change patients with MELD score > 35	Triangular	0.045	-0.021	0.205
Russel Wiesner et al. [15] / Computed	Percent patients with MELD score < 9	Triangular	0.036	0.027	0.045
	Percent patients with MELD score 10–19	Triangular	0.524	0.393	0.655
	Percent patients with MELD score 20–29	Triangular	0.319	0.24	0.399
	Percent patients with MELD score 30–39	Triangular	0.086	0.064	0.107
	Percent patients with MELD score > 40	Triangular	0.035	0.026	0.044
HHS-HRSA-SRTR [14] / Computed	Percent change patients with MELD score < 9	Variable	-0.027	Variable	Variable
	Percent change patients with MELD score 10–19	Variable	-0.003	Variable	Variable
	Percent change patients with MELD score 20–29	Variable	0.022	Variable	Variable
	Percent change patients with MELD score 30–39	Variable	0.034	Variable	Variable
	Percent change patients with MELD score > 40	Variable	0.045	Variable	Variable
HHS-HRSA-SRTR [14] / Computed	Percent change age group 18–34	Fixed	-0.022	-	-
	Percent change age group 35–49	Fixed	-0.068	-	-
	Percent change age group 50–64	Fixed	0.025	-	-
	Percent change age group > 65	Fixed	0.047	-	-
**Theoretical patients’ cohort size and characteristics forecasts (Pre-Transplantation Epidemiology Model)**					
**Reference**	**Parameter**	**Distribution**	**Base**	**Low**	**High**
HHS-HRSA-SRTR [14] / Russel Wiesner et al. [15] / Computed	Mortality and severe sickness rates for patients on waiting-list with MELD score < 9 @ 3-month	Fixed	0.029	-	-
	@ 1-year	Fixed	0.051	-	-
	@ 2-years	Fixed	0.062	-	-
	@ 3-years	Fixed	0.069	-	-
	@ 4-years	Fixed	0.076	-	-
	@ 5-years	Fixed	0.084	-	-
	@ 6-years	Fixed	0.092	-	-
	@ 7-years	Fixed	0.102	-	-
	@ 8-years	Fixed	0.112	-	-
	@ 9-years	Fixed	0.124	-	-
	@ 10-years	Fixed	0.137	-	-
HHS-HRSA-SRTR [14] / Russel Wiesner et al. [15] / Computed	Mortality and severe sickness rates for patients on waiting-list with MELD score 10–19 @ 3-month	Fixed	0.077	-	-
	@ 1-year	Fixed	0.135	-	-
	@ 2-years	Fixed	0.165	-	-
	@ 3-years	Fixed	0.182	-	-
	@ 4-years	Fixed	0.201	-	-
	@ 5-years	Fixed	0.222	-	-
Russel Wiesner et al. [15]	Mortality and severe sickness rates for patients on waiting-list with MELD score 20–29 @ 3-month	Fixed	0.235	-	-
	Mortality and severe sickness rates for patients on waiting-list with MELD score 30–39 @ 3-month	Fixed	0.602	-	-
	Mortality and severe sickness rates for patients on waiting-list with MELD score > 40 @ 3-month	Fixed	0.793	-	-
HHS-HRSA-SRTR [14] / Computed	Percent annual change in time to transplant	Triangular	0.017	-0.017	0.051
HHS-HRSA-OPTN [4]	Waiting time to LT for patients with MELD score < 10 (days)	Triangular	1776	1538	2125
	Waiting time to LT for patients with MELD score 11–18 (days)	Triangular	639	592	698
	Waiting time to LT for patients with MELD score 19–24 (days)	Triangular	106	93	116
	Waiting time to LT for patients with MELD score > 25 (days)	Triangular	20	18	22
	Waiting time to LT for patients with Liver Status 1 (days)	Triangular	6	5	7
HHS-HRSA-SRTR [14] / Computed	Percent of eligible patients not-going for a liver transplant	Uniform	0.213	0.204	0.222
**Theoretical patients’ cohort size and characteristics forecasts (Hospital Admission Epidemiology Model)**					
**Reference**	**Parameter**	**Distribution**	**Base**	**Low**	**High**
Jonathan Showstack et al. [16]	Percent patient required re-transplantation	Fixed	0.03	-	-
	Percent patient died during the index hospitalization	Fixed	0.06	-	-
	Percent patient with UNOS status in hospital but not in ICU	Fixed	0.24	-	-
	Percent patient with UNOS status in ICU	Fixed	0.09	-	-
	Percent donor age 60+ years	Fixed	0.07	-	-
	Percent patient age 60+ years	Fixed	0.19	-	-
	Percent patient with Alcoholic Liver Disease	Fixed	0.2	-	-
	Percent patient with Child-Pugh Class C	Fixed	0.35	-	-
**Theoretical patients’ cohort size and characteristics forecasts (Post-Transplantation Epidemiology Model)**					
**Reference**	**Parameter**	**Distribution**	**Base**	**Low**	**High**
Kenneth Washburn et al. [17] / Milliman Research Report [18] / Jonathan Showstack et al. [16] / CPMC website [19] / Louise Longworth et al. [20]	Post-transplantation survival rate @ Year 1	Fixed	0.90	-	-
	@ Year 2	Fixed	0.85	-	-
	@ Year 3	Fixed	0.79	-	-
	@ Year 4	Fixed	0.76	-	-
	@ Year 5	Fixed	0.74	-	-
	@ Year 6	Fixed	0.71	-	-
	@ Year 7	Fixed	0.69	-	-
	@ Year 8	Fixed	0.67	-	-
	@ Year 9	Fixed	0.65	-	-
	@ Year 10	Fixed	0.63	-	-
John Ammori et al. [21]	First 90 post-operative days frequency of acute cellular rejection	Triangular	0.11	0.11	0.60
	Frequency of biliary complications	Triangular	0.33	0.10	0.33
	Frequency of hepatic artery thrombosis	Triangular	0.03	0.03	0.12
	Frequency of superficial skin infection	Triangular	0.16	0.12	0.20
	Frequency of pneumonia	Triangular	0.16	0.12	0.20
	Frequency of bloodstream infection	Triangular	0.16	0.12	0.20
	Frequency of peritonitis	Triangular	0.17	0.13	0.21
	Frequency of urinary tract infection	Triangular	0.17	0.13	0.21
	Frequency of clostridium difficile colitis	Triangular	0.10	0.08	0.13
	Frequency of other infections	Triangular	0.55	0.41	0.69
	Frequency of venous thromboembolism	Triangular	0.07	0.05	0.09
	Frequency of reoperation	Triangular	0.23	0.17	0.29
	Frequency of primary non-function	Triangular	0.03	0.02	0.04
	Frequency of hepatic vein stenosis	Triangular	0.07	0.05	0.09
	Frequency of acute renal failure	Triangular	0.17	0.13	0.21
**Treatment Costs Model (general)**					
**Reference**	**Parameter**	**Distribution**	**Base**	**Low**	**High**
WHO-GHED [22] / Computed	Annual cost increase rate	Triangular	0.049	0.0258	0.0604
Eugene Yen et al. [12] / David Torgerson and James Raftery [13]	Annual cost discount rate	Triangular	0.03	0.015	0.045
**Pre-Transplantation Treatment Costs Model (costs for 2014-Year 1)**					
**Reference**	**Parameter**	**Distribution**	**Base**	**Low**	**High**
David Axelrod et al. [23] / Adjusted	Average monthly spending in pre-transplantation phase for patients with MELD score < 9	Triangular	$347	$35	$658
	Average monthly spending in pre-transplantation phase for patients with MELD score 10–19	Triangular	$1,578	$1,097	$2,058
	Average monthly spending in pre-transplantation phase for patients with MELD score 20–29	Triangular	$19,602	$13,060	$26,143
	Average monthly spending in pre-transplantation phase for patients with MELD score 30–39	Triangular	$31,644	$20,417	$42,871
David Axelrod et al. [23] / Computed	Average monthly spending in pre-transplantation phase for patients with MELD score > 40	Variable	$51,085	Variable	Variable
Milliman Research Report [18] / Adjusted	Costs adjustment for last 30 days pre-transplantation costs (for patients with MELD score < 30)	Triangular	$26,469	$13,235	$39,704
**Hospital Admission Treatment Costs Model (costs for 2014-Year 1)**					
**Reference**	**Parameter**	**Distribution**	**Base**	**Low**	**High**
Milliman Research Report [18] / Adjusted	Organ procurement costs	Triangular	$73,989	$36,995	$110,984
	Hospital admission costs	Fixed	$330,242	-	-
Jonathan Showstack et al. [16]	Percent of hospital admission costs due to immunosuppressive	Triangular	0.081	0.041	0.121
	Percent of hospital admission costs due to anti-infective	Triangular	0.050	0.025	0.075
	Percent of hospital admission costs due to other medications	Triangular	0.011	0.005	0.016
	Percent of hospital admission costs due to blood products	Triangular	0.120	0.060	0.180
	Percent of hospital admission costs due to operating room	Triangular	0.088	0.044	0.132
	Percent of hospital admission costs due to respiratory services	Triangular	0.041	0.021	0.062
	Percent of hospital admission costs due to special care	Triangular	0.140	0.070	0.210
	Percent of hospital admission costs due to other room and care	Triangular	0.162	0.081	0.243
	Percent of hospital admission costs due to immunosuppressive monitoring	Triangular	0.010	0.005	0.014
	Percent of hospital admission costs due to other laboratory	Triangular	0.145	0.073	0.218
	Percent of hospital admission costs due to chest radiography	Triangular	0.011	0.006	0.017
	Percent of hospital admission costs due to ultrasound	Triangular	0.006	0.003	0.009
	Percent of hospital admission costs due to other imaging	Triangular	0.020	0.010	0.030
	Percent of hospital admission costs due to pathology	Triangular	0.007	0.004	0.011
	Percent of hospital admission costs due to material services	Triangular	0.089	0.045	0.134
	Percent of hospital admission costs due to miscellaneous	Triangular	0.018	0.009	0.027
Milliman Research Report [18] / Adjusted	Physician fees during transplant	Triangular	$48,562	$24,281	$72,843
Jonathan Showstack et al. [16]	Percent increase in costs of treatment for patient requiring re-transplantation	Triangular	1.54	0.77	2.31
	For patient with UNOS status in hospital but not ICU	Triangular	0.15	0.08	0.23
	For patient with UNOS status in ICU	Triangular	0.42	0.21	0.63
	For patient receiving an organ from a 60+ donor	Triangular	0.28	0.14	0.42
	For patient aged 60+ years	Triangular	0.17	0.09	0.26
	For patient with alcoholic liver disease	Triangular	0.26	0.13	0.39
	For patient with Child-Pugh Class C	Triangular	0.41	0.21	0.62
**10-Years Post-Transplantation Treatment Costs Model (costs for 2014-Year 1)**					
**Reference**	**Parameters**	**Distribution**	**Base**	**Low**	**High**
John Ammori et al. [21] / Computed	First 90 post-operative days average costs	Triangular	$139,746	$50,610	$228,882
John Ammori et al. [21] / Adjusted	Additional costs due to acute cellular rejection	Triangular	$21,317	$10,659	$31,976
	Additional costs due to biliary complications	Triangular	$40,457	$20,229	$60,686
	Additional costs due to hepatic artery thrombosis	Triangular	$83,085	$41,543	$124,628
	Additional costs due to superficial skin infection	Triangular	$(2,883)	$(4,325)	$(1,442)
	Additional costs due to pneumonia	Triangular	$59,122	$29,561	$88,683
	Additional costs due to bloodstream infection	Triangular	$75,616	$37,808	$113,424
	Additional costs due to peritonitis	Triangular	$88,187	$44,094	$132,281
	Additional costs due to urinary tract infection	Triangular	$50,609	$25,305	$75,914
	Additional costs due to clostridium difficile colitis	Triangular	$33,939	$16,970	$50,909
	Additional costs due to other infections	Triangular	$50,118	$25,059	$75,177
	Additional costs due to venous thromboembolism	Triangular	$39,148	$19,574	$58,722
	Additional costs due to reoperation	Triangular	$82,231	$41,116	$123,347
	Additional costs due to primary non-function	Triangular	$78,812	$39,406	$118,218
	Additional costs due to hepatic vein stenosis	Triangular	$54,370	$27,185	$81,555
	Additional costs due to acute renal failure	Triangular	$60,766	$30,383	$91,149
CPMC website [19]	Annual cost of immunosuppressive, per patient	Fixed	$36,708	-	-

Table 1 summarizes all the parameters used to perform the epidemiology and treatment cost forecasts with the literature reference or database from which the values were taken. It also shows the distribution and ranges of parameters that were varied to perform the uncertainty and sensitivity analyses.

#### Projection of costs of manufacturing a liver from iPSCs

For the purposes of the manufactured liver cost model, we have made several key assumptions. The initial assumption is that hepatocytes, endothelial cells, and fibroblasts will be sufficient to build an entire liver from scratch. The second assumption is that there will be no proliferation of cells within the bioengineered organ—therefore, every cell will be provided in the transplanted model—which is unlikely, but ensures a conservative model. We have also made the assumption that the cells comprising the bioengineered liver will be purchased as individual cryovials at current catalog list pricing. In this case study, Cellular Dynamics International (CDI) is used as the vendor (http://www.cellulardynamics.com). Factored into the per-vial pricing are the manufacturing costs, royalty payments for IP rights to commercialize, and a profit margin for the vendor.

#### Software

The model is built in Microsoft Excel 2013. Uncertainty and sensitivity analyses are performed with Oracle Crystal-Ball.

## Results

### Epidemiology Model

Assuming the current environment of liver transplantation does not change over the next 20 years, our first set of analyses predicts the total number of new patients on the waiting list will increase from 10,367 new patients in 2014 to 12,763 patients in 2033. This represents a 23% increase in demand for liver transplantation over 20 years. When we compare these figures to the percent of patients with non-identified or non-indicated MELD score on the OPTN database and those with inactive status, the number of new patients on the waiting list with a MELD score is predicted to increase from 7,934 new patients in 2014 to a maximum of 8,006 new patients in 2020, before decreasing to 7,600 new patients in 2033. This decrease does not reflect a decrease in demand for liver transplantation. It reflects the classification of the data reported to the OPTN database and our decision to exclude patients with no reported MELD score. A correction is made to account for those patients in the estimation of potential U.S. liver transplantation demand and the potential U.S. liver transplantation direct medical expenses.

**Fig 2:**
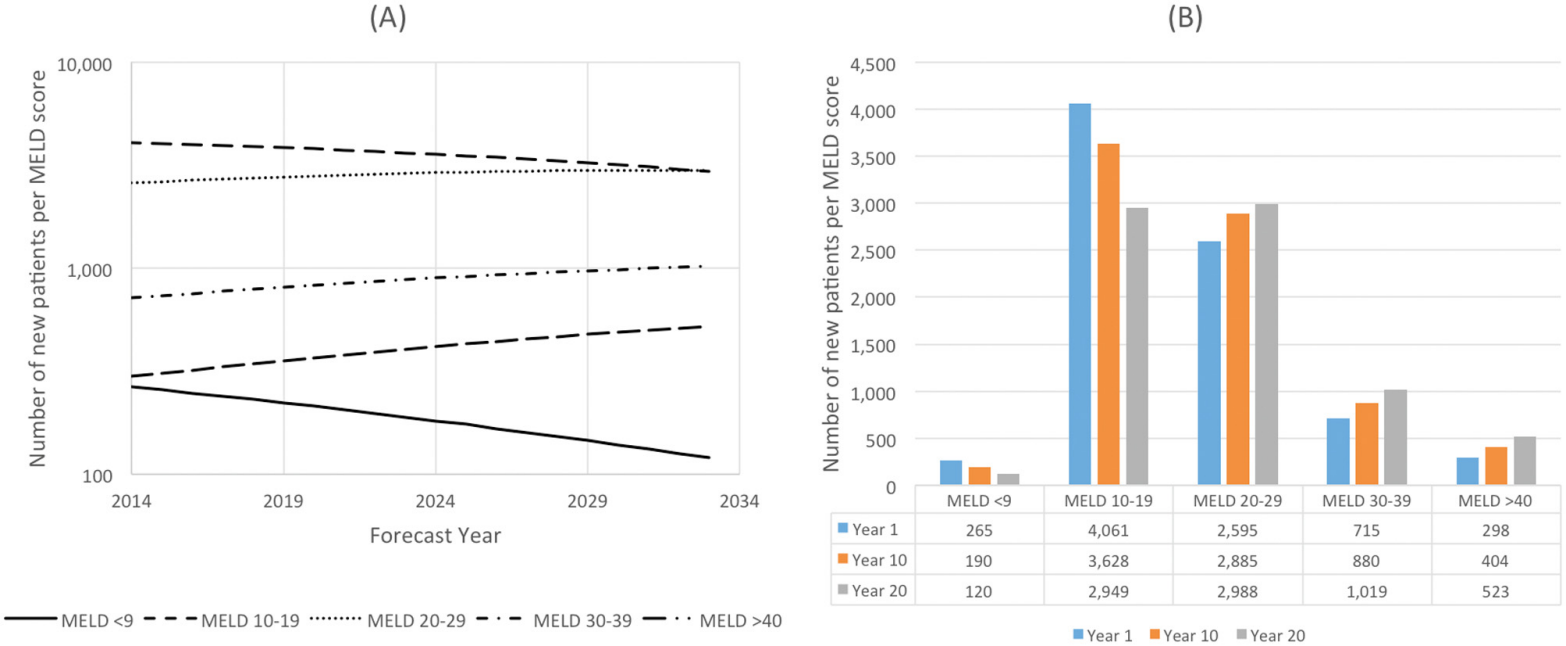
Number of New Patients per MELD score. (A) Metric evolution over 20-years. (B) Metric forecasts at Years 1, 10 and 20.

The forecast of the number of new patients who will be added to the transplant list each year from 2014 to 2033 per MELD score is shown in Fig 2A. These predictions indicate the patients are moving from lower MELD score categories to higher MELD score categories. Between 2014 and 2033 (Fig 2B):

- The number of patients with a MELD score <9 will decrease by 55%
- The number of patients with a MELD score 10–19 will decrease by 27%
- The number of patients with a MELD score 20–29 will increase by 15%
- The number of patients with a MELD score 30–39 will increase by 42%
- The number of patients with a MELD score >40 will increase by 76%

The largest MELD category in 2014 (51%) will be patients scoring 10–19, whereas in 2033 the 20–29 MELD score category will be the largest at 39%. This study predicts that patients' age at listing will increase from an average of 57.7 years in 2014 to an average of 62 years in 2033, a 7% increase.

**Fig 3:**
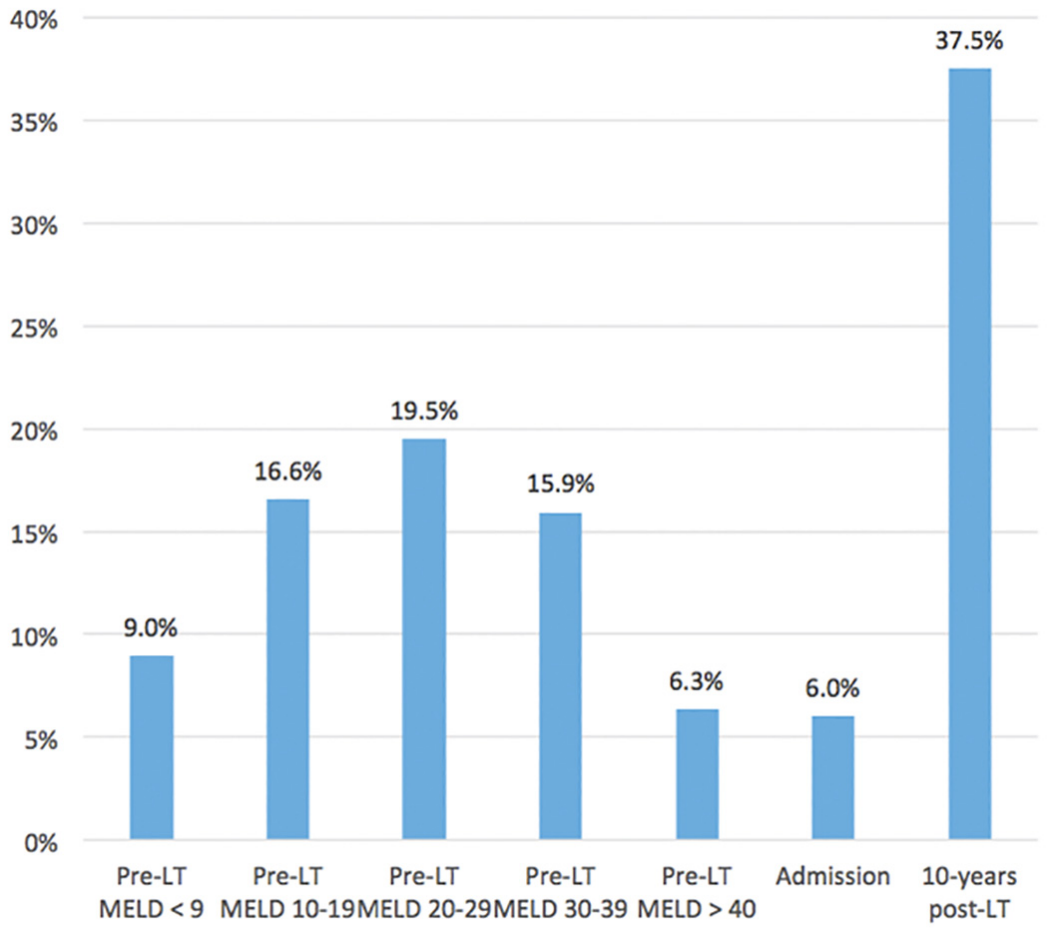
Percent of theoretic patients that would die per liver transplantation phase. The figure shows the predicted mortality percentages. A lower proportion of patients with a high MELD scores (>30), compared to those with a MELD score < 30, are predicted to die due to the significantly shorter wait-time for an organ for sicker patients compared to those in better health.

Based on the OPTN database, this model projects that 65% of the simulated patients over a 20-year period would receive a liver transplant, 18% would not undergo the transplant operation (including patients refusing the operation, patients delisted following improvement in their health conditions, and for other reasons not explicitly stated in the OPTN database). Additionally, the model outcomes predict that 6% of the patients who are operated on would die during hospitalization for transplantation and 63% of transplanted patients would survive the 10-year period following transplantation. Fig 3 provides information regarding the predicted mortality percentages. We also found that a lower proportion of patients with a high MELD scores (>30), compared to those with a MELD score < 30, are predicted to die due to the significantly shorter wait-time for an organ for sicker patients compared to those in better health.

### Treatment Costs Model

**Fig 4:**
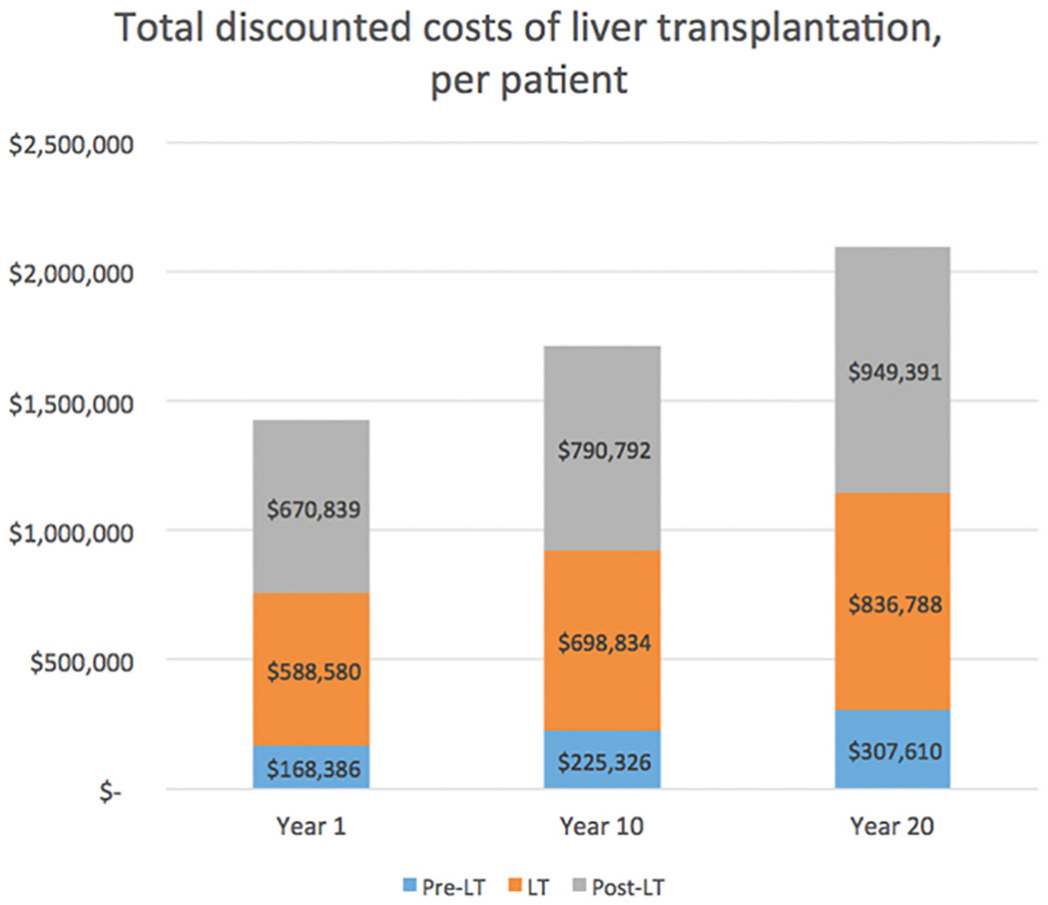
Discounted costs (per theoretical patient and per liver transplantation phase) and total discounted costs of liver transplantation (per theoretical patient) at Years 1, 10 and 20. The total weighted treatment costs of a liver transplantation will increase from $1,427,805 per patient in Year 1 to $2,093,789 per patient in Year 20.

The second set of analyses of forecasting the financial metrics show that the treatment of a patient in the pre-liver transplantation phase will cost between $49,407 (MELD score <9) and $613,020 (MELD score >40) on Year 1. It will increase, respectively, to $71,621 and $867,564 in Year 20. The weighted average treatment costs in the pre-liver transplantation phase are predicted to be $168,386 in Year 1 and will increase by 83% to $307,610 by Year 20. The treatment costs for a patient receiving a liver transplantation (admission phase) and those 10 years post-transplantation will increase by 42% between Year 1 and Year 20 from, respectively, $588,580 to $836,788 and from $670,839 to $949,391 (Fig 4). The total weighted treatment costs of a liver transplantation, all three phases included, will increase from $1,427,805 per patient in Year 1 to $2,093,789 per patient in Year 20 (Fig 4).

**Fig 5:**
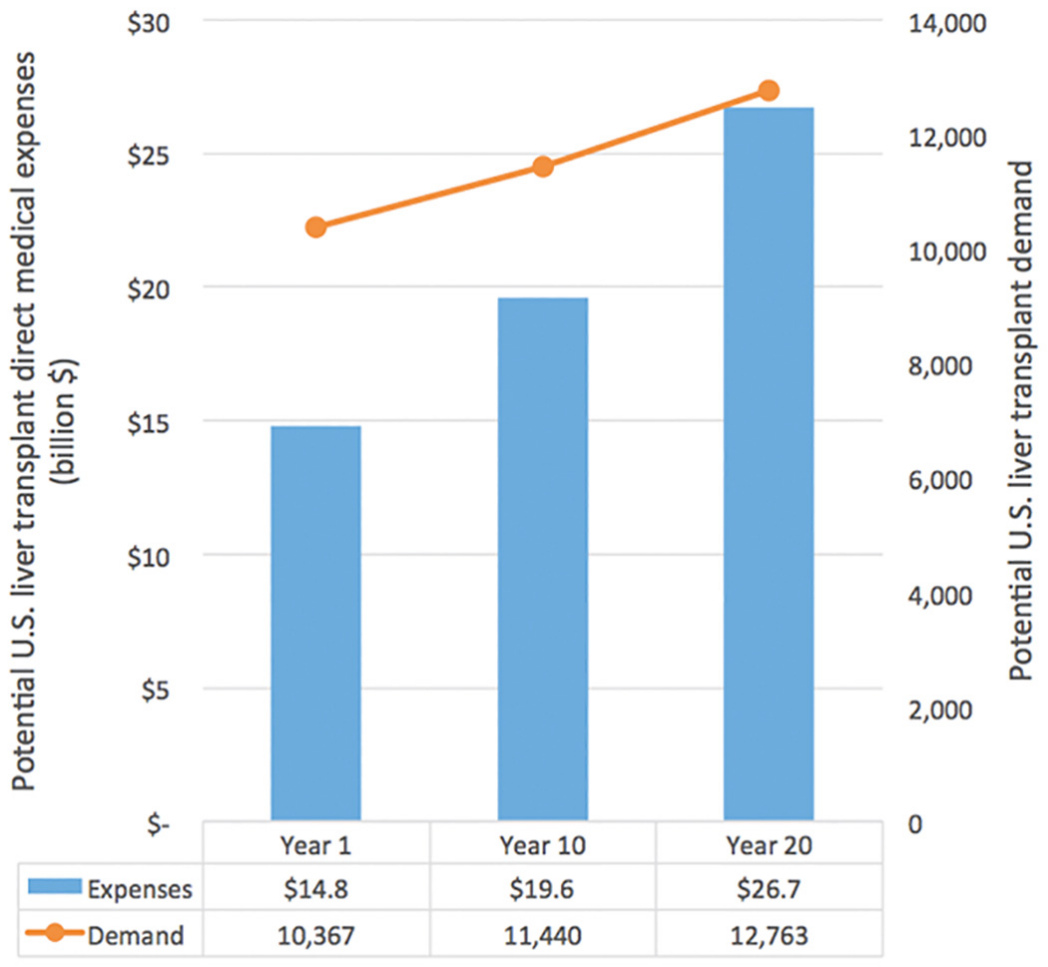
Potential U.S. liver transplant demand and potential U.S. liver transplant medical expenses at Years 1, 10 and 20. The demand for liver organs will increase by 10% in 10 years and by 23% in 20 years.

Fig 5 summarizes the potential U.S. liver transplant demand and the potential U.S. liver transplant medical expenses at Years 1, 10 and 20. Our model predicts that the demand for liver organs will increase by 10% in 10 years and by 23% in 20 years. The potential total costs of liver transplantation (assuming all patients survive the pre-transplantation, admission and 10-year post-transplantation phases) will increase from $14.8 billion to $19.6 billion in 10 years (a 33% increase) and to $26.7 billion in 20 years (an 81% increase).

### Uncertainty Analysis

The uncertainty analysis indicates the *certainty* to achieve the cost projections (at or above), at Year 20, is 36%-39% for the pre-transplant-related costs, 37% for the hospital admission-related costs and 36% for the 10-year post-transplant-related costs (Table 2.). The averages of the 30,000 Monte Carlo iterations are, compared to the base case, 3.6%-5.6% lower for the pre-transplant-related costs and 5.7% lower for the hospital admission-related costs and 10-year post-transplant-related costs. The 10%-90% percentile intervals are as follows:

- Discounted treatment costs in the pre-transplantation phase for patients with MELD score <9: $41,321-$101,380 per patient
- Discounted treatment costs in the pre-transplantation phase for patients with MELD score 10–19: $66,804-$136,787 per patient
- Discounted treatment costs in the pre-transplantation phase for patients with MELD score 20–29: $239,467-$416,221 per patient
- Discounted treatment costs in the pre-transplantation phase for patients with MELD score 30–39: $367,022-$662,244 per patient
- Discounted treatment costs in the pre-transplantation phase for patients with MELD score >40: $592,507-$1,069,105 per patient
- Discounted total costs from day of transplant to discharge: $605,000-$988,004 per patient
- Discounted total costs of treatment 10-years post-transplant: $668,560-$1,143,166 per patient

**Table 2:** Results of the Uncertainty Analysis.

Treatment costs metric	Base case	Certainty to base case	Average	Median	10% Percentile	90% Percentile
Discounted treatment costs in pre-LT phase for patients with MELD score < 9, @ Year-20 listing, per to-be-transplanted patient	$71,621	39%	$68,870	$65,009	$41,321	$101,380
Discounted treatment costs in pre-LT phase for patients with MELD score 10–19, @ Year-20 listing, per to-be-transplanted patient	$103,002	39%	$99,293	$95,655	$66,804	$136,787
Discounted treatment costs in pre-LT phase for patients with MELD score 20–29, @ Year-20 listing, per to-be-transplanted patient	$342,614	36%	$323,679	$317,667	$239,467	$416,221
Discounted treatment costs in pre-LT phase for patients with MELD score 30–39, @ Year-20 listing, per to-be-transplanted patient	$537,402	37%	$507,360	$497,873	$367,022	$662,244
Discounted treatment costs in pre-LT phase for patients with MELD score > 40, @ Year-20 listing, per to-be-transplanted patient	$867,564	37%	$819,501	$803,749	$592,507	$1,069,105
Discounted total resources used from day of transplant to discharge, @ Year-20 listing, per transplanted patient	$836,788	37%	$789,166	$779,109	$605,000	$988,004
Discounted total costs of treatment 10-years post-liver transplantation, @ Year-20, per operated patient	$949,391	36%	$895,228	$881,537	$668,560	$1,143,166

An uncertainty analysis is used to assess the accuracy of the projections made, giving the percentage at which it’s likely to achieve (or exceed) the projected (base) value. (Example: the costs of hospital admission period are likely to be higher than the projections in 37% of the cases.) The uncertainty analysis also gives the averages (certainty equal to 50%) and the confidence intervals (the most likely values ranging from 10% to 90% percentiles).

### Sensitivity Analysis

The sensitivity analysis revealed that all treatment costs model outputs, at Year 20, are sensitive to the annual cost increase (sensitivity index 24%-54%) and the annual cost discount rate (sensitivity index -17% to -28%) (Table 3). Pre-transplantation-related costs, per MELD category, are also sensitive to the average monthly spending in the pre-transplantation phase (except for MELD score 10–19; sensitivity index 22% to 42%), the last 30-days pre-transplantation costs (only for MELD score <9; sensitivity index 12%), and the percent in annual change in time to transplant (only MELD scores <9 and 10–19; sensitivity index 22% to 40%).

**Table 3:** Results of the Sensitivity Analysis.

Treatment costs metric	Sensitivity Analysis	
	Input variable	Index
Discounted treatment costs in pre-LT phase for patients with MELD score < 9, @ Year-20 listing, per to-be-transplanted patient	Annual cost increase	24%
	Percent annual change in time to transplant	22%
	Average monthly spending in pre-LT phase for patients with MELD score < 9	22%
	Cost discount rate	-17%
	30 days pre-LT costs	12%
Discounted treatment costs in pre-LT phase for patients with MELD score 10–19, @ Year-20 listing, per to-be-transplanted patient	Percent annual change in time to transplant	40%
	Annual cost increase	26%
	Cost discount rate	-18%
Discounted treatment costs in pre-LT phase for patients with MELD score 20–29, @ Year-20 listing, per to-be-transplanted patient	Annual cost increase	39%
	Average monthly spending in pre-LT phase for patients with MELD score 20–29	33%
	Cost discount rate	-28%
Discounted treatment costs in pre-LT phase for patients with MELD score 30–39, @ Year-20 listing, per to-be-transplanted patient	Average monthly spending in pre-LT phase for patients with MELD score 30–39	42%
	Annual cost increase	34%
	Cost discount rate	-24%
Discounted treatment costs in pre-LT phase for patients with MELD score > 40, @ Year-20 listing, per to-be-transplanted patient	Average monthly spending in pre-LT phase for patients with MELD score 30–39	42%
	Annual cost increase	34%
	Cost discount rate	-24%
Discounted total resources used from day of transplant to discharge, @ Year-20 listing, per transplanted patient	Annual cost increase	54%
	Cost discount rate	-39%
Discounted total costs of treatment 10-years post-liver transplantation, @ Year-20, per operated patient	Annual cost increase	54%
	Cost discount rate	-39%

A sensitivity analysis assesses which model input has most influence on the model output, meaning a small change in that specific input value will result in high variation in the model output. Other model inputs might have a moderate influence (an input variation that would result in a comparable variation in the output) or low influence (meaning even a high variation in the input wouldn’t affect the output). A sensitivity analysis is designed to select the most sensitive inputs, for which high precision and accurate information are required.

### Projection of Costs to Manufacture Liver Grafts from iPSCs

The manufacture and universal availability of a liver graft from autologous tissue and cells would change the paradigm of organ transplantation by increasing the number of organs available for transplantation, and eliminate the need for life-long immune suppression and its complications. Several strategies are being developed for construction of whole organs using the decellularized whole organ matrix, and subsequent re-seeding with relevant cell types, in physiologically appropriate bioreactors [24–30]. Engineering pluripotency of human somatic cells by the ectopic expression of transcription factors has opened the possibility of generating autologous cells for cell/organ replacement therapies. Thus, iPSCs are of special interest because they could be patient specific, can be propagated indefinitely as undifferentiated cells and can differentiate into practically any cell type [31].

In Table 4 we show the cost projections estimated based on the number of iPSC-derived hepatocytes and iPSC-derived endothelial cells along with donor-derived fibroblasts (not currently a CDI catalog product) needed to populate the entire liver and represent a base case estimate representative of the high end of projected costs [32–34]. Included in the cost model in Table 4 is the list price associated with reprogramming primary tissue to iPSCs, which is relatively insignificant. Most of the cost is associated with hepatocyte production. However, although reprogramming itself is not a major contributor to cost, selecting an optimal clone for production can require a considerable amount of expertise. In some instances of end-stage liver disease, and for inborn errors of liver metabolism, an entire liver may not be required and auxiliary partial liver transplantation has been suggested as a therapeutic option [10]. For this reason, we’ve also projected the costs of manufacturing only 35% and 15% of a liver.

**Table 4:** Projection of Costs to Manufacture a Liver from iPSCs.

Material / Effort	Cells per liver	Cells per vial	Vials per liver	Cost per vial	Cost per liver	Cost for 35% of liver	Cost for 15% of liver
Reprogramming (non GMP)					$15,000	$15,000	$15,000
Hepatocytes	1.50E+11	9.00E+06	1.67E+04	$1,500	$25,000,000	$8,750,000	$3,750,000
Endothelial cells (40% of HCs)	6.00E+10	3.20E+07	1.88E+03	$800	$1,500,000	$525,000	$225,000
Fibroblasts (10% of HCs)—cells per vial/cost (*estimate)*	1.50E+10	1.00E+07	1.50E+03	$800	$1,200,000	$420,000	$180,000
Total					$27,715,000	$9,710,000	$4,170,000

## Discussion

The simple truth is that significantly more livers are needed for transplantation. Transplantation is the definitive treatment for end-stage liver disease, yet one in ten people on the transplant waiting list die because of the dearth of organs. Additionally, the number of individuals on the waiting list gauges only a fraction of the true need. According to clinicians, thousands more could benefit from a transplant but are precluded from the waiting lists because they have not yet reached critical status. The shortfall of organs is predicted to rise in the coming decades given the increasing prevalence of liver-damaging viruses, a surge in fatty liver disease, and a buildup of environmental toxins.

The social need for greater availability of livers for transplant is bolstered by an economic argument. The epidemiology and treatment costs models forecast demand for liver transplantation will increase by 10% in 10 years and by 23% in 20 years. Total weighted treatment costs of a liver transplantation—including pre-operative, admission and post-operative phases—will increase from $1,427,805 per patient in Year 1 to $2,093,789 in Year 20 (Fig 4).

This study is subject to certain limitations with the major challenge being access to databases. To build and run the model, data was collected from different sources and databases available online (Table 1) which may have introduced errors in computing and forecasting the epidemiology and financial metrics. The result of studies relying on retrospective data may also be compromised by potential coding errors and incompleteness [35]. Additionally, as a consequence of the sparse data, the impact of the MELD score on treatment costs is only evaluated in the pre-liver transplant phase.

Moreover, our model excludes several new developments that are likely to modify the course of liver transplantation. Currently, the top four causes of chronic liver disease in patients on the U.S. liver transplant waitlist are chronic hepatitis C virus (HCV) infection, alcoholic liver disease (ALD), nonalcoholic steatohepatitis (NASH), and a combination of chronic HCV infection and ALD (HCV/ALD). However, these rankings may change. New direct-acting antiviral agents are transforming the treatment of chronic hepatitis C. A recently approved single-tablet regimen of the hepatitis C virus (HCV) NS5A inhibitor ledipasvir and the NS5B inhibitor sofosbuvir (ledipasvir/sofosbuvir; Harvoni) has been reported highly effective [36–38]. However, it is an expensive therapy and cost may represent a barrier to treatment [39]. Also additional studies have demonstrated a lower than expected prevalence of chronic HCV in the U.S., raising the concern the decrease is secondary to HCV-related deaths [40]. Meanwhile, NASH increased 170 percent, making it the second leading etiology of chronic liver disease among new liver transplant waitlist registrants in 2013. During the same period, new waitlist registrants with ALD increased 45 percent, and registrants with chronic HCV infection increased 14 percent [41]. The number of patients with NASH awaiting liver transplantation is anticipated to continue climbing, while availability of donor organs is expected to decline. Combined, these factors reveal the changing epidemiology of patients awaiting liver transplantation in the U.S.

Additionally, several novel cellular therapies to induce tolerance in solid-organ transplant patients have entered early-phase clinical trials [42]. Although immunosuppression is necessary to prevent immune attacks on the transplanted organ, it also imposes substantial morbidity and mortality risks for transplant recipients. Whether these novel cellular therapies aimed at improving organ tolerance will have a significant impact on the organ donor pool will have to be determined.

However, despite these limitations the financial outcomes forecasted in Year 1 in our model are in similar ranges as those reported by Buchanan [43] and co-workers (2009) for 990 patients ($77,100 with MELD score 6–14 to $237,300 with MELD score 28–40) for the period one-year pre-transplant; $267,200-$332,200, respectively, for the transplant admission period; and $71,300 to $88,000, respectively for the period one-year post-transplant [43].

From the results of Buchanan et al [43] one should note that the MELD score has a higher impact on treatment costs in the pre-transplant period compared to treatment costs of transplant admission and post-transplant periods. Also, high variability exists in the costs reported by the authors, with the coefficient of variation exceeding 100% in many cases (97% to 166% for the pre-transplantation-related costs; 70% to 76% for the hospital admission-related cost; and 150% to 211% for the one-year post-transplantation costs) [43]. If it's generally recognized that the MELD score is a driver of costs in liver transplantation, the latter result shows that other drivers exist and should be identified and accounted for.

Liver allocation in the U.S. is donation-based with the sickest patients prioritized using the Model for End-Stage Liver Disease (MELD) score. Following implementation of the MELD system, the number of patients who died while on the waiting list fell, but the health status of listed patients deteriorated. Care of wait-listed patients is expensive. The cost of treating severely ill patients, reflected by a high MELD score, requires longer hospital admissions, more frequent and longer use of Intensive Care Units, and more laboratory tests and medications [43].

Further complicating the problem, organ availability is limited not only by the number of donors, but also by the inability to convert *eligible* donor organs into *actual* donor organs. Schnitzler et al (2005) [44] have suggested that, due to the lack of identification of prospective donors and the difficulty in obtaining consent, the eligible-donor/actual-donor conversion rate is estimated to be just 42%. This represents an annual loss of 250,000 life-years in the U.S. alone. New measures have been employed to counter the organ shortage. The cold ischemia time (the time from aortic clamping in the donor to the time of removal from ice prior to transplantation) has been increased from 6 hours to more than 18 hours [45], and organs from donors with a high Donor Risk Index are being used [46]. Still, the shortage of livers for transplantation persists.

Social policy may ameliorate the shortage in the short term. Adopting an opt-out system, for example, will make all citizens automatic donors unless they choose to opt out and will certainly increase the availability of organs. This will put the eligible-donor/actual-donor conversion rate at 100% and presumably will not fall below the current rate. Additionally, creating a marketplace for organs has proponents, but this proposed solution raises numerous ethical concerns, generating considerable opposition. Neither an opt-out system nor establishing a marketplace for organs is a likely probable solution.

The shortage may be overcome by the manufacture of bioengineered livers from autologous stem cells. Elements of the technology, although not yet validated by a reproducible protocol, are in place. Critical aspects of the developmental biology of the liver remain unknown but the manufacture of replacement organs is no longer science fiction. Relatively soon anyone needing a liver could have one bioengineered from one of their own skin cells. Because there will be no immune reaction, there will be no need for immunosuppressive drugs, an added clinical and financial benefit over using a cadaveric organ.

How far are we from a bioengineered organ? The factors that drive the self-organization of the cells comprising the liver are not yet fully detailed. The question becomes, do we need to recapitulate all the evolutionary programming that drives cells to form metazoan structures? Perhaps there are shortcuts. That’s the promise of decellularized scaffolds [47]. Will decellularization provide the basis of the protocol to bioengineer a liver? Or, will it simply be a platform for studying the self-organization of the hepatocytes and endothelial cells that comprise a liver? It remains to be seen.

Blood or skin cells from a person with a high MELD score are already the sources of iPSCs from which hepatocyte precursor cells are made. In an *in vitro* or *in vivo* bioreactor, or by means of bioprinting, these hepatocyte precursor cells will provide the building blocks for a customized liver that originates from patient-specific somatic cells.

Certainly the current tremendous cost ($9.7M) to bioengineer just 35% of a liver—a tissue mass that may be sufficient to maintain critical metabolic function—is not a viable option. However, these costs will decrease dramatically as this therapeutic approach gains traction. Why? Because the bulk of the manufacturing cost is associated with cell production and these costs will fall with economies of scale.

The time required for reprogramming and selecting a clone for production can be reduced by banking personal iPSCs (comprehensive iPSC banking will likely be a critical component of healthcare in the future), which places the focus on reducing the costs associated with multiple growth and differentiation media used in the manufacturing process.

Reagents comprised of growth factors and small molecules are used to activate and/or repress signaling pathways at key checkpoints that determine cellular fate. Pulling these molecular levers at the precise moments necessary to drive well-defined tissue specification across the appropriate germ layers of endoderm, ectoderm and mesoderm requires knowledge of the signaling mechanisms affecting gene regulation and protein signaling cascades.

Technicians begin the cell-production process upstream of differentiation with the initial culture of the stem cells. Stem-cell culture is typically effected in mTeSR or the more cost efficient and defined E8 media, both of which are composed of growth factors and signaling molecules. These media provide stable stem cell cultures in the absence of a feeder layer, minimizing variability and maintaining the cells in a state of readiness to enter differentiation.

There are multiple hepatocyte differentiation protocols. All follow the same approximate timeline of 25–30 days and employ similar induction factors [48–51] (bFGF and Activin A, HGF, Oncostatin M, Dexamethasone, etc.). Reagents comprise over 50% of current costs for differentiation from iPSCs, so achieving cost improvements here will be critical to the goal of manufacturing a liver. Beyond the issue of cost of differentiation, differentiated cells derived from iPSCs may still be immature, functionally resembling a fetal or neonatal phenotype. Achieving the appropriate cellular maturity may prove to be critical for most kinds of liver failure. This is a realistic expectation given the stem-cell field is still in its adolescence and will continue to expand at an accelerated rate over time. High-volume demand for affordable reagents to support manufacture of differentiated tissue will drive economies of scale.

In addition to the inevitable reduction in production costs of reagents, there will be corresponding efficiencies in cell production that will increase batch sizes and reduce labor costs depending on the choice of bioreactor (defined as any production mechanism outside of flask culture used to differentiate and expand the cells). Bioreactors could be envisioned as *in vivo* (large animal), or recellularized organ tissue scaffolds that could be maintained *in vivo*, *ex vivo* or as a *de novo* engineered scaffold used in combination with a bioreactor device. Any of these approaches could lead to improvements in production scale and concomitant cost reductions.

The cost of sequencing the first human genome was approximately $300 million [52, 53]. (Factor in all the ground-laying research funded through government programs over the preceding 15 years and the price tag exceeds $3 billion [52].) Today, a human genome can be sequenced in a few days for a cost of $3,000-$5,000 [54].

Given the potential to benefit from numerous cost-saving measures—lower reagent costs, improved production system technology, increased scale, and the use of earlier stage progenitor cells—future cell-manufacturing costs will certainly be a fraction of current estimates. It follows that the cost of bioengineering replacement livers for transplant will be financially feasible in the not too distant future.

Nevertheless, organ-engineering technology is in its infancy and will need to overcome countless translational hurdles. For instance, complete reestablishment of the liver micro-architecture would require incorporation of liver nonparenchymal cells (e.g. bile duct cells, sinusoidal endothelial cells, stellate cells, etc), necessitating iPSC differentiation for these cell types. Additionally, differentiated cells derived from iPSCs may still be immature, functionally resembling fetal or neonatal phenotype. Cell maturity may prove to be critical for diseases requiring functional differentiated cells. So although proof-of-principle for whole-organ assembly and transplantation has been shown for three of the solid organs (heart, liver, lungs), numerous obstacles must be overcome before the cells generated can be used widely in preclinical studies. Perhaps other innovative technologies already on the horizon (e.g. organ reprogramming) may change the future of liver transplantation [55–57].

## Supporting Information

S1 Economic Model A 20-Year Epidemiology Model and Treatment Costs Model for Liver Transplantation. (XLSX)
